# Validity and Reproducibility of a Spanish EPIC Food Frequency Questionnaire in Children and Adolescents

**DOI:** 10.3390/nu16223809

**Published:** 2024-11-07

**Authors:** Ana Larroya, María Tamayo, María Carmen Cenit, Yolanda Sanz

**Affiliations:** 1 Microbiome, Nutrition & Health Research Group, Institute of Agrochemistry and Food Technology, Spanish National Research Council (IATA-CSIC), 46980 Valencia, Spain; analarroya@iata.csic.es (A.L.); mcenit@iata.csic.es (M.C.C.); yolsanz@iata.csic.es (Y.S.); 2 Department of Medicine, Autonomous University of Madrid, 28029 Madrid, Spain

**Keywords:** dietary assessment, nutrient intake, FFQ, 24H-DR, reproducibility, validity, children, adolescents, EPIC

## Abstract

Background: Dietary habits are crucial for preventing many diseases, particularly in children and adolescents. Accurate assessment of dietary intake is essential for understanding the relationship between diet and health in these age groups. Objective: This study aimed to evaluate the reproducibility and validity of a Spanish version of the European Prospective Investigation into Cancer and Nutrition (EPIC) Food Frequency Questionnaire (FFQ) in 150 Spanish children and adolescents aged 10 to 17 using the average of 9 days of 24-h dietary recall (24H-DR) as a reference. Methods: Intraclass correlation coefficients (ICCs) were calculated to asses reproducibility and Spearman/Pearson correlation coefficients were calculated to assess validity and reproducibility. Results: The average ICCs were 0.41 for crude nutrients, 0.31 for food groups, 0.31 for energy-adjusted nutrients, and 0.4 for energy-adjusted food groups. Spearman/Pearson correlation coefficients averaged 0.39 and 0.41 for crude and energy-adjusted nutrients, respectively, and 0.51 and 0.47 for corresponding food groups. Regarding validity, the average correlation coefficient for crude, energy-adjusted, and de-attenuated nutrients was 0.32, 0.50, and 0.50, respectively. The highest crude coefficient was 0.50 for vitamin C and fiber, while the highest energy-adjusted coefficient was 0.76 for protein and carbohydrates. The highest de-attenuated coefficient was 0.72 for vitamin B6. Conclusions: Overall, these results suggest that the EPIC FFQ is a valid and reliable instrument for assessing dietary intake in Spanish children and adolescents.

## 1. Introduction

Childhood and adolescence are important developmental periods characterized by physical and mental changes. During this vulnerable phase, lifestyle factors, including dietary intake, physical activity, and nutritional status, play a pivotal role in preventing the onset of several diseases [1]. Poor dietary habits, in particular, can contribute to the onset of chronic conditions such as obesity, type 2 diabetes, cardiovascular disease, and nutritional deficiencies, which may increase the risk of neurodevelopmental disorders such as attention-deficit-hyperactivity disorder (ADHD) [2,3]. This highlights the urgent need for effective and reliable tools to measure and assess dietary characteristics in children and adolescents [2,4]. Common dietary assessment tools include the 24-h dietary recall (24H-DR), food records (FRs), weight food records (WFRs), food frequency questionnaires (FFQs), and, less frequently, blood or urine biomarkers of specific nutrients [5]. Each method has its own strengths and weaknesses, and the optimal choice depends on factors such as the size of the study population, economic constraints, research objectives, and the target population.

FFQs are dietary assessment tools that ask respondents to indicate how often they have consumed various foods and beverages over the last year. These questionnaires typically include between 5 and 227 food items with specified portion sizes and offer 9 frequency response options [5,6]. FFQs are practical, easy to administer, and inexpensive, making them ideal for assessing long-term dietary intake in large populations [7]. They are commonly used in nutrition research to estimate food groups and nutrient intakes and to classify individuals by dietary patterns. Due to their simplicity and cost-effectiveness, FFQs are the most widely used tools in epidemiological nutrition for studying relationships between dietary intake and health outcomes, including metabolic, mental, and immunological conditions [2,6,8,9,10]. In the Spanish population, validity and reproducibility studies for FFQs have focused primarily on adults [11,12,13,14,15], with relatively few studies on preschool [16,17] and school-aged [18] children. Moreover, only a limited number of dietary assessment tools have been found to be both reproducible and valid in children and adolescents [4,5,18,19]. Validity studies in these populations have used small sample sizes and have not investigated the relationship between dietary risk factors and diseases.

The European Prospective Investigation into Cancer and Nutrition (EPIC) is a comprehensive study examining the links between diet, nutritional status, lifestyle, and environmental factors and the incidence of cancer and other chronic diseases across several European countries [15]. One valuable resource from this study is the EPIC Norfolk Food Consumption Questionnaire (FFQ), which covers a wide range of dietary items related to immunity, genetic predisposition, and environmental factors that contribute to inflammatory processes. This makes it an excellent tool for assessing dietary intake in younger populations. While the EPIC FFQ has been validated for use in Spanish adults [15,20,21], its reliability and validity for Spanish children and adolescents has not been established. To address this gap, we developed and adapted a Spanish version of the EPIC FFQ and evaluated its performance in this younger population. Once its reliability is confirmed, this adapted version can be used to investigate the relationship between nutrition and health.

## 2. Materials and Methods

### 2.1. Study Population and Design

**Figure 1:**
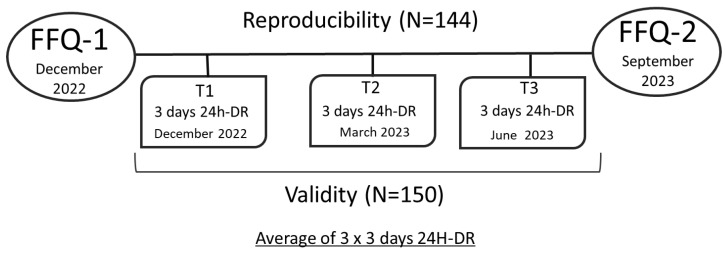
Timeline of questionnaire collection.

This was a longitudinal study designed to evaluate the validity and reproducibility of the EPIC FFQ adapted to Spanish language to assess dietary intakes of children and adolescents aged 10–17 years. Participants (*n* = 250) were recruited from the general population of two different schools located in Valencia (Spain) and excluded those with eating behavior problems or highly fluctuating dietary habits. To assess variability, we asked participants during each 24H-DR if their reported consumption differed from their typical eating patterns on other days. Data from the EPIC FFQ were collected twice: in December 2022 (FFQ1) and September 2023 (FFQ2). For the validity study, three 24H-DRs were collected on three non–consecutive days at three different points throughout the six-month study, and the average of these served as the reference [5,8]. A total of nine 24H-DRs were utilized as the reference method. The first 24H-DR (T1) was gathered in December together with the FFQ1, the second (T2) in March 2023, and the last (T3) in June 2023. Figure 1 shows a scheme of the study timeline. The study was approved by the Ethics Committee of the Spanish National Research Council (CSIC) on 3 of May 2022 (Reference 056/3 May 2022). Children, adolescents, and their parents gave written informed consent.

### 2.2. EPIC-Norfolk Food Consumption Frequency Questionnaire

The EPIC FFQ is a 137-item self-administered semi-quantitative food consumption frequency questionnaire [15]. It was translated into Spanish using a language understandable to children and adolescents and was adapted for online completion using LimeSurvey Community Edition Version 6.6.1+240806 (Hamburg, Germany: LimeSurvey GmbH n.d.). Participants accessed the questionnaire via a digital link.

The FFQ collects annual consumption data for each food through 9 consumption frequency responses: never or less than once, 1–3 times a month, 1 time per week, 2–4 times per week, 5–6 times per week, 1 time per day, 2–3 times per day, 4–5 times per day, and more than 6 times a day. To standardize and estimate portion sizes of the different food items referred to in the questionnaire, participants were shown the ENALIA photographic atlas (developed by the Spanish Agency for Food Safety and Nutrition (AESAN) [22]. An additional questionnaire gathered sociodemographic (sex and age), anthropometric (weight and height), physical activity, and special diet use (e.g., gluten-free or vegetarian) information. Two trained nutritionists assisted with questionnaire completion (correct interpretation and training in completing the questionnaires) as well as in the use of the ENALIA photographic atlas. A total of 20 food group were evaluated, while the daily intakes of energy and 36 macro- and micronutrients were calculated using the National Higher Nutrition and Dietetics Education Center food tables (CESNID) [23], Spanish food composition databases (RedBEDCA) [24], and the United States Department of Agriculture (USDA) nutrition and food composition tables [25].

### 2.3. Three-Day 24-h Dietary Recall (24H-DR)

Information from three non-consecutive days (including one day of the weekend and two working days) was gathered to use the 24H-DR as the reference method. The first three 24H-DRs were completed concurrently with the FFQ1 in December 2021; the second set of three 24H-DRs were completed three months later in March 2022; and the third set of three 24H-DRs were completed in June 2022. Two trained nutritionists explained to volunteers how to complete the 24H-DR and use the ENALIA photographic atlas [22].

Daily nutrient intake and energy data from 24H-DR were calculated using Dietopro dietary and nutritional management software (URL: https://dietopro.com, accessed on 17 July 2024) [26], and the same 36 macro- and micronutrients as in the EPIC FFQ were also assessed.

### 2.4. Statistical Analysis

IBM SPSS Statistics software (version 27.0) was used for statistical analysis. *p* values < 0.05 were considered statistically significant. The normality of the data distribution was evaluated using the Kolmogorov–Smirnov test. Quantitative variables were expressed as the mean and standard deviation (SD) or the median and interquartile range (IQR). Categorical variables were analyzed using the chi-square test and presented as percentages. To assess reproducibility, we calculated Spearman’s rank correlation coefficient (r) for non–normally distributed data and Pearson’s correlation coefficient for normally distributed data, after transformation of the values to their natural logarithm (ln), to compare energy, nutrients, and food groups between the FFQ1 and FFQ2. Values were interpreted as: r > 0.50 good, 0.2 < r < 0.49 acceptable, and r < 0.2 poor [27]. The reliability of the EPIC FFQ was assessed using the test–retest method based on comparing the responses collected in FFQ1 and FFQ2 by calculating the interclass correlation coefficient (ICC) with a confidence interval of 95%. Values were interpreted as ICC > 0.75, good reliability; 0.75 < ICC < 0.4, moderate reliability; and ICC < 0.4, poor reliability [28]. All evaluations were conducted using crude and energy-adjusted variables obtained through the residual method as advised by Willet [8]. This approach ensures that the values are not influenced by total energy intake, controlling the variability of dietary intake and reducing over- or underestimation bias. Finally, to evaluate the percentage of agreement between FFQ1 and FFQ2, we used misclassification and quartile agreement method.

To evaluate the validity of the EPIC FFQ, we compared the FFQ2 and the 24H-DR data. We calculated Spearman’s or Pearson rank correlation coefficient (r) after transforming the values to their natural logarithm (ln). The criteria considered were the same as those in the reproducibility study. All analyses were carried out using crude, energy-adjusted, and de-attenuated coefficients. De-attenuated correlation coefficients were evaluated to eliminate random error caused by intra- and inter-person variability in the three 24H-DRs. Thus, we used the formula from Rosner and Willet [29]: rt = r0 √1 + r (e σw2/σb2)/n; where r0 is the observed correlation between FFQs and 24H-DR; r is the rate of variation within- and between-person measured during three 24H-DRs; and n is the number of days of dietary recall (*n* = 3). We employed Bland–Altman plots to examine the agreement between the two types of dietary assessment across intake levels. A natural-log (ln) transformation was applied to refine the 95% limits of agreement (LOAs). Visualization of the limits of agreement (LOAs) (ln mean difference ± 1.96 SD) between the methods was performed by plotting the difference between the FFQ2 and the three 24H-DRs against the mean of the two methods [30].

## 3. Results

A total of 250 children and adolescents from two different schools in Valencia (Spain) were recruited. Of these, 150 (60%) correctly completed all the questionnaires for the validity study, while 144 (57.6%) participated in the reproducibility study. The characteristics of the population included in the validity study are presented in Appendix A Table A1. The participants were almost evenly distributed between males and females (52.6% and 47.4%, respectively), with a mean age of 14.3 years (SD = 2.0). Most participants (75.3%) had an optimal weight, while 24.7% had overweight or obesity, with a mean weight of 58.0 kg (SD = 13.3), a mean height of 164.5 cm (SD = 12.6), and a mean body mass index (BMI) of 21.1 kg/m^2^ (SD = 3.2), all of which were calculated based on the z-score curves established by the World Health Organization (WHO) for school-aged children and adolescents [31]. In terms of education level, eight classrooms participated, including one primary (10–11 years old), five secondary (12–15 years old), and two high-school (16–17 years old) classes. Regarding levels of physical activity, 31.3% of participants reported low levels, 44.7% moderate levels, and 24% high levels of activity. Statistically significant sex-related differences were observed only in weight levels (*p* < 0.001) and sports participation (*p* < 0.001).

### 3.1. Reproducibility

As noted previously, 144 people were included in the reproducibility study. When the EPIC FFQ was administered at two different time points, significant differences in daily nutrient intakes (g/day) and food group intakes (g/day) were observed (See Table 1 for nutrients and Appendix A Table A2 for food groups). In general, the FFQ1 reported a higher intake for several items, including energy, total protein, animal protein, vegetable protein, total fats, monounsaturated fatty acids (MUFAs), polyunsaturated fatty acids (PUFAS), saturated fatty acids (SFAs), cholesterol, carbohydrates, sodium, iron, calcium, selenium, zinc, magnesium, phosphorus, iron, zinc, selenium, vitamin B1, vitamin B6, vitamin B3 or niacin, vitamin B9 or folates, vitamin A, and sugar confectionary. Contrastingly, the FFQ2 reported higher consumption of fresh fruit, refined cereals, whole grain cereals, and processed meat. These differences could be attributed to seasonal variations or reflect both changes in participants’ eating behaviors and variations in data recording.

To assess the reproducibility of the EPIC FFQ, we evaluated whether similar results were obtained when the measurements were repeated at two time points (FFQ1 and FFQ2) under similar conditions through different types of analysis. Table 2 shows the ICCs and Spearman and Pearson correlation coefficients between the two sets of data for crude and energy-adjusted nutrients, and Appendix A Table A3 shows the ICCs and correlation coefficients for the food groups. The ICCs of crude nutrient intake between the two FFQs ranged from 0.18 for vitamin D to 0.54 for energy; the ICCs of the crude food group intake ranged from 0.15 for pastries to 0.66 for processed meat. After adjusting for energy, the ICCs of nutrient intake ranged from 0.08 for iron to 0.51 for vitamin C, and the ICCs of food group intake ranged from 0.11 for cheese to 0.60 for vegetables. The average ICCs of crude and energy-adjusted nutrient intake were 0.41 and 0.31, respectively. The average ICC was 0.40 for both the crude food groups and after adjustment for energy. Accordingly, we observed moderate reliability between the FFQ1 and FFQ2 for crude nutrients and food groups but poor reliability for nutrients when we adjusted for energy.

With regards to the Spearman and Pearson coefficients, we observed that for crude nutrients, the correlation coefficients ranged from 0.19 for iron to 0.52 for vitamins B1 and B6. The correlation coefficients for crude food groups ranged from 0.30 for eggs and vegetable fats to 0.74 for processed meat. After adjusting for energy, the correlation coefficients for nutrient intake ranged from 0.14 for iron to 0.50 for vitamin C; and for food group intake, they ranged from 0.21 for eggs and sauces and processed food to 0.67 for vegetables and processed meat. The average correlation coefficients of the crude and energy-adjusted nutrients were 0.39 and 0.41, respectively. The average correlation coefficient of crude food group intake was 0.51, while the average correlation coefficient of energy-adjusted food group intake was 0.47. Consequently, between the FFQ1 and FFQ2, we observed acceptable consistency for crude nutrients and energy-adjusted nutrients and food groups, and good consistency for crude food groups.

To measure the percentage of agreement between the FFQ1 and FFQ2, we used misclassification rates and adjacent quartile agreement to measure the proportion of participants who were classified in different or in the same quartile in the two measurements by the same FFQ. The best agreement percentages between the FFQ1 and FFQ2 were 85.4% for vitamin B1 and 89.4% for white meat. A misclassification into the opposite quartile >10% indicated low agreement between two FFQ assessments. The results showed that all foods and most nutrients had correct values (<10%) with the exception of iron (10.4%) (See Appendix A Table A4).

### 3.2. Validity

A total of 150 participants were included in the validity study (See Appendix A Table A1). We used the FFQ2, which captures dietary intake over the past 6 months, for comparison with the information collected during the same period using the 24H-DR.

We found differences between the FFQ2 and 24H-DR questionnaire with regards to nutrient intake (g/day). The FFQ2 recorded higher intakes of energy, sodium, iodine, SFAs, cholesterol, potassium, magnesium, phosphorus, iron, selenium, zinc, vitamins B1, B2, B12, B9, C, and A, total fats, PUFAs, MUFAs, and total fiber than the 24H-DR (Table 3). This may suggest that the FFQ2 overestimates intake levels.

Table 4 summarizes the crude, energy-adjusted, and de-attenuated Pearson correlation coefficients of nutrient intake between the FFQ2 and 24H-DR. The attenuation correction was applied to eliminate intra- and interindividual variations. The Pearson correlation coefficient for crude nutrients ranged from 0.19 for SFAs and PUFAs to 0.50 for vitamin C and fiber. The coefficients of nutrients adjusted for energy ranged from 0.25 for SFAs and folate to 0.76 for total protein and carbohydrates. The de-attenuated Pearson correlation coefficient ranged from 0.30 for SFAs to 0.72 for vitamin B6. As expected, stronger correlations were observed for energy-adjusted nutrients and de-attenuated coefficients. The average correlation coefficients of the crude and adjusted-energy nutrients and the de-attenuated coefficient were 0.32, 0.50, and 0.50, respectively. Therefore, acceptable consistency was observed for crude nutrients between the FFQ and 24H-DR, while good consistency was observed when adjusting nutrients for energy and using de-attenuated coefficients.

Furthermore, we assessed the percentage of agreement between the FFQ2 and the 24H-DR questionnaire using misclassification rates and adjacent quartile agreement (See Appendix A Table A5). Once nutrients were divided into quartiles, the agreement percentage in the same or adjacent quartile between the FFQ2 and 24H-DR ranged from 65.56% for cholesterol and SFAs to 83.44% for vitamin C; the average percentage agreement for nutrient intake was 72.46%. Misclassification into the opposite quartile was <10% for all nutrients with the exception of total fats (10.60%).

**Figure 2:**
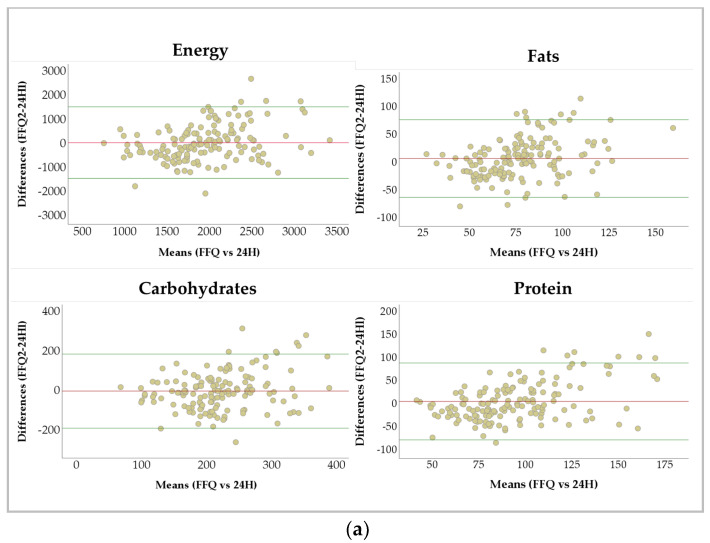

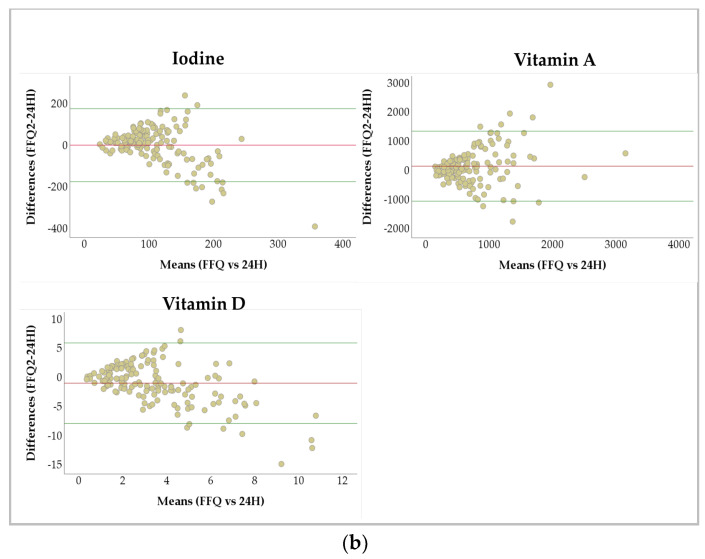
Bland–Altman plots showing the relationship between mean and differences in daily nutrient intake estimated with the average of FFQs and three 24H-DRs. (**a**) Energy, fats, protein and carbohydrates show good concordance. (**b**) Iodine, vitamin A and vitamin D did not show a good correlation. Red lines show the mean of the differences and green lines represent the Limits of Agreement (LOA).

**Figure A1:**
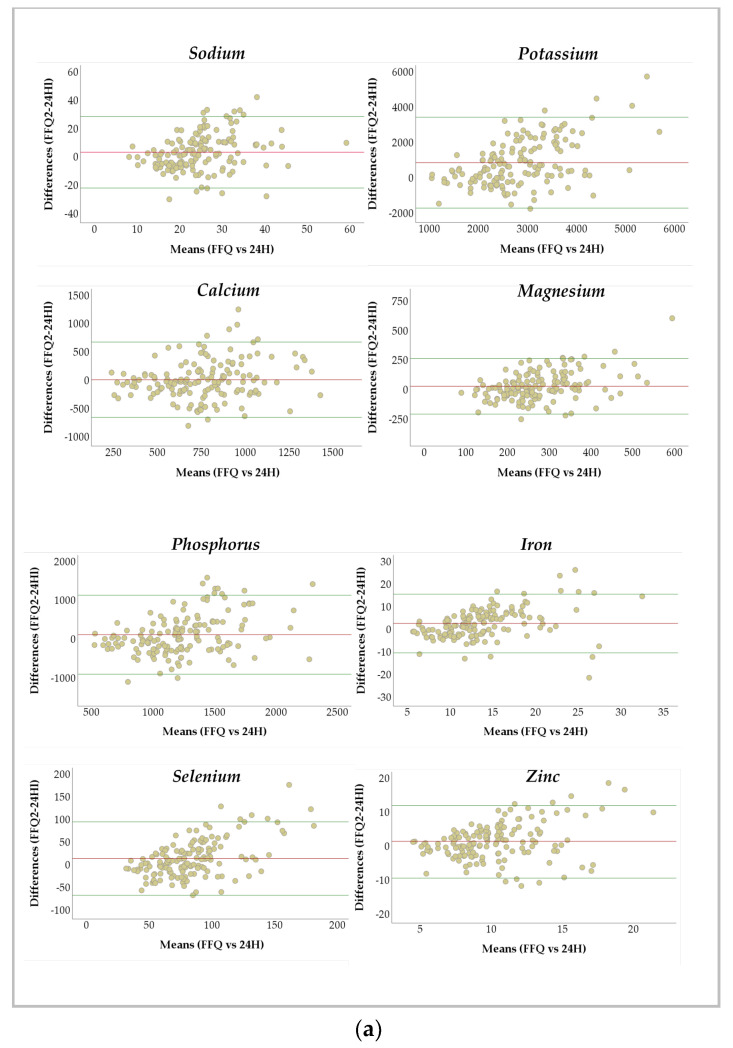

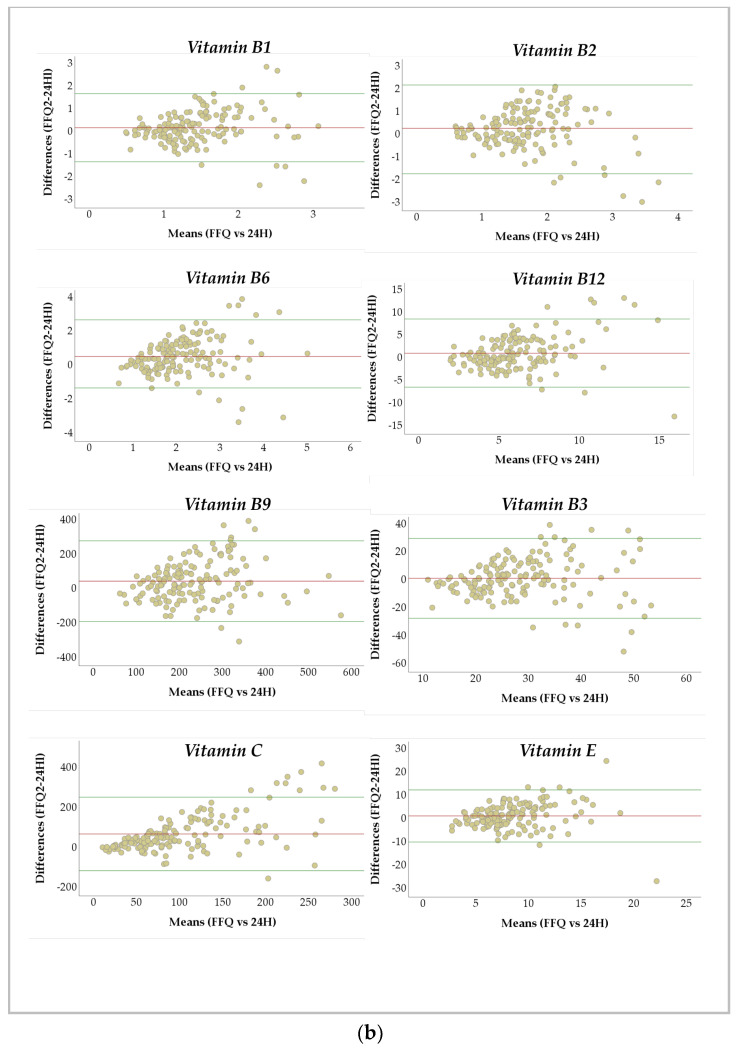

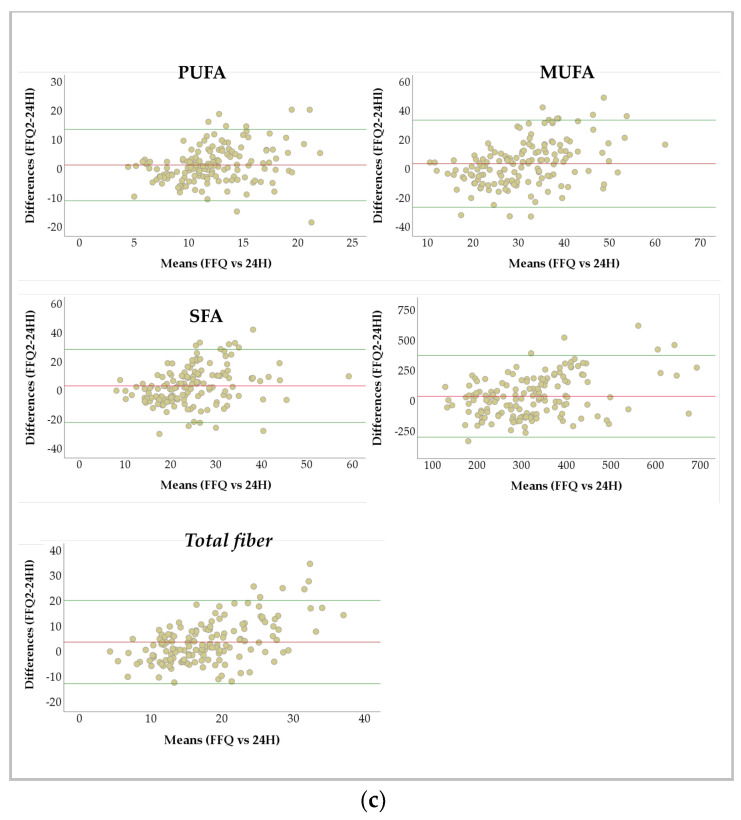
Bland–Altman plots showing the relationship between the means and differences in the daily nutrient intake estimated with the average of the FFQs and three 24H-DRs. (**a**) Plots showing sodium, potassium, calcium, magnesium, phosphorus, iron, selenium, and zinc. (**b**) Vitamin B1, B2, B6, B12, B9 or folate, B3 or niacin, C, and E. (**c**) Plots showing saturated fatty acids (SFAs), polyunsaturated fatty acids (PUFAs), monounsaturated fatty acids (MUFAs), cholesterol, and fiber. Red lines show the mean of the differences and green lines represent the Limits of Agreement (LOA).

Bland–Altman analysis was performed to quantify the agreement between the FFQ2 and 24H-DR. Table 4 shows that all nutrients remained within the LOA; less than 10% of subjects had values outside the LOA for all nutrients. Bland–Altman plots of energy and macronutrient intakes are shown in Figure 2a. These plots help to assess the agreement between the two methods of measuring dietary intake. Most of the data points closely aligned with the average line, with the exception of iodine, vitamin A, and vitamin D (Figure 2b), which reflects good agreement between the two questionnaires. The remaining nutrient data are shown in Appendix A (See Appendix A Figure A1).

## 4. Discussion

The present study validates the Spanish-adapted EPIC FFQ in children and adolescents aged 10 to 17 years old, providing a reliable dietary assessment tool for this population. Compared with other methods such as FRs, WFRs, and biomarkers, 24H-DR is considered the most practical reference method for this age group due to its simplicity and ease of use [6,8]. To account for individual and stationary variations, we collected the three 24H-DRs three times over six months, spanning different seasons of the year. The sample sizes for the reproducibility and validity studies were 144 and 150, respectively, which are considered optimal [6]. The combination of extensive 24H-DR collection and large sample sizes has demonstrated strong validity and study quality [6].

In terms of reproducibility, we observed an overestimation of most nutrients (Refer to Table 1) and some food groups (Refer to Table A2) in the FFQ1, consistent with the findings of Rendo-Urteaga et al. [9]. The average ICC was 0.40 for nutrients and food groups. When we adjusted for energy, the ICC decreased to 0.31 for nutrients (Refer to Table 2), while it remained similar for food groups (Refer to Table A3). This decrease has been reported previously, which implies errors in the estimation of energy intake or variability in the subject’s response [18,32,33]. Our findings are consistent with the results reported in previous studies involving children and adolescents where the correlation coefficient and ICC averages ranged from 0.3 to 0.5 [18,34,35]. However, other studies in this population observed higher correlation coefficients and ICCs, ranging from 0.5 to 0.9 [9,36,37]. These high correlations can likely be explained by the much shorter interval between the administrations of the two FFQs, ranging from two weeks to one month. By contrast, the nine-month gap in our study allows for seasonal dietary changes, potentially resulting in lower correlations [6,8]. Nevertheless, our results demonstrated acceptable reliability for nutrients and food groups, excluding vitamin D and iron.

We achieved a high level of agreement for nutrient (78.2%) and food group (81.9%) intake (Refer to Table A4), matching or surpassing previous studies [18,38]. This demonstrates that our EPIC FFQ is a reliable and valid tool for collecting and assessing dietary intake in Spanish adolescents and older children, aligning with established criteria [4,5,6,8,39].

Regarding validity, we found higher intake estimations for most nutrients from the FFQ2 compared with 24H-DR, with the exception of calcium, vitamin B6, vitamin B3, vitamin D, vitamin E, and carbohydrates (refer to Table 3). As reported in previous studies in children and adolescents, the FFQ tends to overestimate nutrient intake [18,35,38,40,41,42,43,44,45]. Inge Huybrechts et al. [21] suggest that this could be attributed to differences in data collection and questionnaire structure. It could also be due to the lack of collection of intakes from certain food groups by 24H-DR and difficulties in estimating portion sizes [21].

In the correlation analysis, we observed minor differences in Spearman and Pearson correlation coefficients, and, therefore, only Pearson’s correlation was computed to adjust for total energy intake and intra- and inter-individual variation. The average crude intake was 0.32 (refer to Table 4), aligning with findings from similar studies [38,45] but exceeding those reported in others [18,35,46]. Upon adjusting for energy, the correlation increased to 0.5 (refer to Table 4). As expected, the stronger correlations when adjusting for energy were likely due to the close relationship between inter-subject intake variability and total energy intake [21]. The average de-attenuated correlation coefficient was 0.45 (refer to Table 4), reflecting an improvement when compared with crude intakes. This is likely attributed to intra- and inter-individual correction, as reported by Willet and Rosner [8,29]. The German EPIC study on FFQ reproducibility and validity also demonstrated that adjusting for attenuation due to within-person error in the reference method leads to more accurate and reliable correlation measurements [47].

The percentage of participants classified into the same or an adjacent quartile for nutrient intake was higher (72.46%) than that reported in a recent similar study [18] and equal to previous studies in the same population type [32,38,44,48]. Total fats were the only nutrient for which 10% or more of the children were classified into the opposite quartile (Refer to Table A5).

The Bland–Altman plots revealed no substantial bias in macronutrients and energy assessments between the two methods (refer to Figure 2a). For micronutrients, the EPIC FFQ seems to overestimate vitamin A, vitamin D, and iodine intake compared with 24H-DR, showing proportional bias (refer to Figure 2b). The Bland–Altman index agreement showed a correct classification of all nutrients (<10%) (refer to Table 4). It is important to note that Bland–Altman plots provide only a visual interpretation [30], and their findings should be considered in conjunction with correlation coefficients and cross-classification into quartiles [18]. According to our results, both methods are in good agreement with each another.

The strengths of the present study include a suitable sample size (more than 100 participants), providing sufficient statistical power for dietary validation [3]; the use of 24H-DR as the reference method, which is the best available and most recommended method; the inclusion of data from three different time points of 24H-DR days, including a weekend day to have more representative data of the habitual diet, and the time interval between the different 24H-DR recording points captured seasonal variations; and the use of a photographic atlas to aid in food recognition and portion sizes. Limitations of the study include a lengthy questionnaire (137 food items) compared with other FFQs (fewer than 100), which may lead to participant fatigue and over-reporting of certain foods, potentially affecting accuracy [8]. While the long study duration (9- to-12-month data collection period) can be considered a strength regarding seasonality, it may also increase the likelihood of diet changes, potentially affecting reproducibility and validity. Although participants were trained and provided with a photographic food atlas, self-administration of the EPIC FFQ and 24H-DR may introduce some misreporting due to forgetfulness or difficulty recalling dietary intakes. Finally, the longer recall period of the FFQ over the 24H-DR may increase the risk of recall bias, leading to memory issues and potential inaccuracies [4].

## 5. Conclusions

The Spanish-adapted EPIC FFQ has been validated for all nutrients and demonstrated reproducibility for both food groups and nutrients in a large sample of Spanish children and adolescents. The online version of the questionnaire, which was developed and evaluated in this study, is more user-friendly and adaptable to the target population. The study design, which included questionnaires administered at different times of the year, confirms the reliability of the FFQ, including its ability to capture seasonal variations in dietary intake. However, further research is needed to develop new, shorter, and more accurate methods to assess dietary intake in this population. Overall, our results suggest that the Spanish-adapted EPIC FFQ is a suitable method for assessing dietary intake in Spanish older primary school children and adolescents, which constitutes important progress in the availability of dietary assessment tools at crucial stages of development and in the understanding of the relationship between diet and health.

## Figures and Tables

**Table 1:** Nutrient intake of the population in the reproducibility study (*n* = 144).

Nutrients	FFQ1						FFQ2						*p* ^‡^
	M *	Med *	SD *	25	50	75	M	Med	SD	25	50	75	
Energy (kcal)	2508.4	2328.7	1199.1	1720.4	2328.7	2878.6	2119.1	1974.1	1112.3	1399.8	1974.1	2529.3	<0.001
Total protein (g)	128.9	113.5	82.6	88.3	113.5	141.2	105.4	102.5	32	80.4	102.5	126.1	0.03
Animal protein (g)	87.3	78.7	59.9	58.9	78.7	98.7	74.1	69.3	30.7	50.9	69.3	93.4	0.014
Vegetable protein (g)	41.6	36.7	28	28.7	36.7	44.7	31.4	30	9.5	24.1	30	37.3	<0.001
Total fats (g)	110.5	96.6	78.2	74.7	96.6	121.4	86.7	85.5	21.7	70.9	85.5	103.3	0.04
MUFAs (g)	44.3	38.9	32.3	27.6	38.9	49.5	35.3	34.6	10.2	28	34.6	42.6	0.02
PUFAs (g)	18.8	16.3	13.5	12.3	16.3	20.6	14	13.7	4.2	10.8	13.7	16.5	<0.001
SFAs (g)	40.2	35.1	29.5	26.9	35.1	44.5	28.5	27.9	7.9	22.9	27.9	34.9	<0.001
Cholesterol (mg)	440.3	364.6	316	306.5	364.6	482.8	369.1	358.5	131.5	271.2	358.5	441.9	0.02
Carbohydrates (g)	316.2	277.4	203.3	236.2	277.4	324.9	236.3	229.4	57.7	187.4	229.4	284.8	<0.001
Total fiber (g)	28.6	25.2	19.7	18.4	25.2	32.9	22	20.2	9.1	15.3	20.2	25.7	0.03
Iodine (µg)	128.8	114.2	88.5	84.8	114.2	143.9	119.2	111.6	40.9	91.1	111.6	140.1	0.82
Sodium (mg)	3296.9	2948.9	2235.2	2220.2	2948.9	3592.9	2469.1	2286.4	750.5	1956.8	2286.4	3049.2	<0.001
Potassium (mg)	4350.9	3869.9	2851.1	2883.8	3869.9	4801.9	3551.4	3437.5	1001.8	2758.8	3437.5	4157.1	0.06
Calcium (mg)	1116.4	964	733.7	728.5	964	1283.9	831.2	800.7	284.8	643.6	800.7	977.4	<0.001
Magnesium (mg)	527.9	429.2	359.5	361.6	429.2	588.8	311.2	300.9	88.8	249.6	300.9	375.6	<0.001
Phosphorus (mg)	1759.1	1536.6	1142	1245.9	1536.6	1865.1	1409.1	1387.5	355.3	1127.5	1387.5	1661.3	0.03
Iron (mg)	30.4	24.4	21.2	19.1	24.4	32.9	16	15.9	4.6	12.4	15.9	18.9	<0.001
Zinc (mg)	15.3	13.4	10.2	11.1	13.4	16.6	12	11.4	3.4	9.2	11.4	14.1	<0.001
Selenium (µg)	127.5	113.8	80.3	90.8	113.8	140.1	97.5	94.8	31.9	72.9	94.8	114.1	<0.001
Vitamin B1 (mg)	2	1.7	1.2	1.4	1.7	2.2	1.7	1.6	0.5	1.2	1.6	2	0.03
Vitamin B2 (mg)	2.2	1.9	1.4	1.6	1.9	2.4	2	1.9	0.5	1.5	1.9	2.3	0.29
Vitamin B6 (mg)	3.1	2.7	2	2.2	2.7	3.5	2.6	2.5	0.8	2	2.5	3.1	0.002
Vitamin B12 (µg)	7.9	7.2	5.1	5.4	7.2	9	7.2	6.6	2.9	5.2	6.6	8.6	0.3
Vitamin B9 (µg)	370.1	318.5	251.4	235.6	318.5	416.6	281.2	262.9	113.8	198.1	262.9	350.8	<0.001
Vitamin B3 (mg)	42.5	38.1	27.4	30.5	38.1	44.7	31.6	31.5	9.9	24.3	31.5	37.9	<0.001
Vitamin C (mg)	222.7	171.3	193.2	98.8	171.3	274.2	148.8	124.3	95.2	74.9	124.3	207.9	<0.001
Vitamin A (µg)	1141.5	875.3	1067.5	592.9	875.3	1368.4	837.6	652.9	542.3	448.3	652.9	1088.2	<0.001
Vitamin D (µg)	3.3	2.6	2.8	1.5	2.6	4.1	3.4	2.8	2.1	1.9	2.8	4.5	0.51
Vitamin E (mg)	12	10.7	8.6	7.3	10.7	13.7	9.9	9.4	3.8	6.9	9.4	12.5	0.1

* Intakes are expressed as the mean (M), standard deviation (SD), median (Med), and interquartile range (P25:P75). ^‡^ *p*-values of median differences were calculated using the Wilcoxon test.

**Table 2:** Reliability and consistency of nutrient intake between the FFQ1 and FFQ2.

Nutrients	ICCs		Pearson or Spearman Coefficient	
	r ^a^	r-Adjusted ^b^	r ^c^	r-Adjusted ^d^
Energy (kcal)	0.538 (0.44–0.465)	0.538 (0.44–0.465)	0.50	0.5
Total protein (g)	0.430 (0.290–0.560)	0.332 (0.180–0.470)	0.41	0.47
Animal protein (g)	0.511 (0.380–0.620)	0.447 (0.306–0.579)	0.44	0.43
Vegetable protein (g)	0.323 (0.169–0.482)	0.219 (0.058–0.369)	0.32 *	0.44
Total fats (g)	0.397 (0.250–0.526)	0.220 (0.060–0.371)	0.31	0.38
MUFAs (g)	0.412 (0.270–0.540)	0.244 (0.084–0.391)	0.32	0.32
PUFAs (g)	0.428 (0.285–0.550)	0.273 (0.115–0.420)	0.35	0.38
SFAs (g)	0.385 (0.240–0.515)	0.280 (0.122–0.424)	0.34	0.46
Cholesterol (mg)	0.398 (0.251–0.530)	0.243 (0.084–0.391)	0.35	0.38
Carbohydrates (g)	0.354 (0.203–0.490)	0.20 (0.028–0.342)	0.39	0.24 *
Total fiber (g)	0.414 (0.269–0.540)	0.342 (0.190–0.479)	0.41 *	0.48
Iodine (µg)	0.290 (0.137–0.436)	0.287 (0.130–0.430)	0.34	0.42
Sodium (mg)	0.359 (0.208–0.493)	0.239 (0.079–0.387)	0.37	0.38
Potassium (mg)	0.436 (0.290–0.560)	0.314 (0.159–0.454)	0.48	0.48
Calcium (mg)	0.394 (0.237–0.536)	0.280 (0.124–0.425)	0.32	0.40
Magnesium (mg)	0.310 (0.155–0.451)	0.234 (0.074–0.383)	0.32	0.32
Phosphorus (mg)	0.375 (0.225–0.507)	0.304 (0.149–0.445)	0.39	0.37 *
Iron (mg)	0.237 (0.070–0.385)	0.083 (−0.08–0.243)	0.19	0.14
Zinc (mg)	0.451 (0.311–0.572)	0.358 (0.207–0.492)	0.43	0.51
Selenium (µg)	0.384 (0.236–0.515)	0.302 (0.146–0.443)	0.38	0.36
Vitamin B1 (mg)	0.499 (0.366–0.612)	0.404 (0.258–0.532)	0.52	0.53
Vitamin B2 (mg)	0.399 (0.252–0.528)	0.345 (0.193–0.481)	0.33	0.51
Vitamin B6 (mg)	0.523 (0.394–0.632)	0.386 (0.238–0.517)	0.52	0.41 *
Vitamin B12 (µg)	0.433 (0.290–0.556)	0.387 (0.239–0.517)	0.45	0.46
Vitamin B9 or folate (µg)	0.460 (0.321–0.580)	0.380 (0.231–0.511)	0.48	0.47
Vitamin B3 or niacin (mg)	0.504 (0.374–0.616)	0.306 (0.150–0.447)	0.48	0.33
Vitamin C (mg)	0.514 (0.383–0.624)	0.428 (0.285–0.552)	0.5	0.46
Vitamin A (µg)	0.478 (0.341–0.595)	0.407 (0.267–0.534)	0.44	0.45
Vitamin D (µg)	0.178 (0.015–0.331)	0.178 (0.015–0.331)	0.23	0.20
Vitamin E (mg)	0.446 (0.305–0.568)	0.343 (0.190–0.479)	0.45 *	0.38
Average	0.41	0.31	0.39	0.41

FFQ1 vs. FFQ2 after 10 months. ICC: intra-class correlations coefficient. ^a^ r: ICC after nutrient or food group crude intake was transformed to the natural logarithm (ln). ^b^ r-adjusted: after nutrient intake or food group intake was adjusted for energy using the residual method. ^c^ r: correlation coefficient after nutrient or food group crude intake was transformed to the natural logarithm (ln). ^d^ r-adjusted: correlation coefficient after nutrient intake or food group intake was adjusted for energy using the residual method. * Pearson correlation.

**Table 3:** Nutrient intake of the population in the validity study (*n* = 150).

Nutrients	3 × 3 Days 24H-DR					FFQ2					*p* ^‡^
	M *	SD *	Med *	P25 *	P75 *	M	SD	Med	P25	P75	
Energy (kcal)	1929.59	545.28	1892.47	1549.48	2200.15	2201.77	1288.6	1974.08	1412.54	2552.59	0.01
SFAs (g)	22.59	8.67	20.74	16.73	26.98	29.76	19.34	25.9	17.12	36.24	<0.001
Sodium (mg)	2192.87	754.41	2117.61	1658.8	2624.44	2532.67	1570.23	2161.81	1564.33	3108.67	0.01
Cholesterol (mg)	298.85	113.6	282.33	228.7	370.4	387.12	269.83	323.5	231.89	443.29	<0.001
Iodine (µg)	116.3	164.1	76.58	53.81	130.53	122.75	79.63	104.59	78.6	139.05	0.04
Potassium (mg)	2376.05	803.39	2278.82	1802.05	2855.08	3668.65	2424.79	3149.14	2283.77	4283.86	<0.001
Calcium (mg)	761.93	350.19	726.82	553.12	943.66	853.48	521.62	760.03	523.02	1015.57	0.18
Magnesium (mg)	268.79	165.12	249.86	202.5	303.94	326.19	208.21	269.11	204.39	382.06	<0.001
Phosphorus (mg)	1196.65	424.51	1154.05	926.83	1357.61	1443.95	826.76	1309.99	919.52	1664.39	<0.001
Iron (mg)	12.92	8.54	11.21	9.38	14.13	16.93	10.46	14.39	10.46	20.65	<0.001
Selenium (µg)	79.44	48.68	73.65	59.64	88.14	100.65	60.53	85.08	60.19	120.32	<0.001
Zinc (mg)	10.02	7.79	8.75	7.01	11.29	12.41	8.1	10.74	7.69	13.97	<0.001
Vitamin B1 (mg)	1.52	2.02	1.22	0.95	1.64	1.71	1.13	1.4	0.99	2.13	<0.001
Vitamin B2 (mg)	1.8	2.6	1.43	1.03	1.82	2	1.15	1.83	1.28	2.44	<0.001
Vitamin B6 (mg)	2.46	5.29	1.64	1.32	2.19	2.67	1.75	2.26	1.61	3.07	0.25
Vitamin B12 (µg)	5.96	4.49	5.19	3.9	7.18	7.52	5.48	6.34	4.25	8.7	<0.001
Vitamin B9 (µg)	242.27	299.99	193.82	144.08	258.71	296.94	230.89	242.98	174.08	354.24	<0.001
Vitamin B3 (mg)	31.68	31.66	24.93	20.06	33.15	32.53	19.99	28.62	19.5	38.13	0.08
Vitamin C (mg)	77.5	144.77	52.41	30.39	84.69	161.59	179.82	113.16	64.09	200.43	<0.001
Vitamin A (µg)	722.74	1194.4	495.93	304.3	831.4	926.93	890.56	616.4	393.15	1106.67	<0.001
Vitamin D (µg)	5	9.89	2.92	1.17	5.82	3.48	3.3	2.91	1.69	4.4	0.14
Vitamin E (mg)	10.91	24.71	7.17	5.57	9.09	10.34	7.55	8.44	5.87	12.71	0.42
Carbohydrates (g)	222.36	73.74	217.04	171.83	264.58	242.76	139.39	216.4	156.61	284.4	0.06
Total protein (g)	91.96	27.33	90.47	74.2	106.27	108.58	68.34	94.5	65.19	124.5	0.001
Total fats (g)	72.87	22.72	70.07	57.03	83.69	89.17	52.5	82.17	55.66	103.91	<0.001
PUFA (g)	11.32	4.27	10.82	8.66	13.36	14.21	8.03	12.66	9.04	17.19	<0.001
MUFA (g)	28.87	9.47	27.94	21.58	34.73	36.56	22.43	32.04	22.36	44.78	<0.001
Total fiber(g)	16.01	5.99	15.72	12.07	19.04	23.24	19.35	18.39	13.53	26.38	<0.001

* Intakes are expressed as the mean (M), standard deviation (SD), median (Med), and interquartile range (P25:P75). ^‡^ *p*-values of the median differences were calculated using the Wilcoxon test and *p* values of the mean were calculated using a paired *t*-test.

**Table 4:** Validity of FFQ2 nutrient intake compared with 24H-DR and Bland–Altman agreement.

Nutrients	Spearman			Bland–Altman Index (%) ^‡^
	r ^a^	r-adj ^b^	r de-att ^c^	
Energy (kcal)	0.374		0.44	4
SFAs (g)	0.19	0.25	0.3	8
Sodium (mg)	0.29	0.69	0.58	4.7
Cholesterol (mg)	0.21	0.46	0.36	4
Iodine (µg)	0.29	0.37	0.39	7.3
Potassium (mg)	0.32	0.65	0.38	3.3
Calcium (mg)	0.39	0.59	0.47	4.7
Magnesium (mg)	0.4	0.64	0.46	4
Phosphorus (mg)	0.33	0.7	0.39	6.7
Iron (mg)	0.4	0.58	0.49	8
Selenium (µg)	0.38	0.56	0.49	4
Zinc (mg)	0.2	0.59	0.31	6
Vitamin B1 (mg)	0.39	0.64	0.46	4.7
Vitamin B2 (mg)	0.36	0.51	0.53	4
Vitamin B6 (mg)	0.39	0.32	0.72	6
Vitamin B12 (µg)	0.28	0.32	0.5	5.3
Vitamin B9 or folate (µg)	0.37	0.25	0.46	4.7
Vitamin B3 or niacin (mg)	0.36	0.44	0.5	8
Vitamin C (mg)	0.5	0.27	0.58	6.7
Vitamin A (µg)	0.39	0.39	0.48	6
Vitamin D (µg)	0.235	0.4	0.55	6
Vitamin E (mg)	0.20	0.3	0.5	4
Carbohydrates (g)	0.31	0.76	0.38	6
Total protein (g)	0.33	0.76	0.39	5.3
Total fats (g)	0.2	0.67	0.31	6
PUFAs (g)	0.19	0.4	0.33	5.3
MUFAs (g)	0.24	0.5	0.36	6.7
Total fiber (g)	0.5	0.6	0.56	5.3
Average	0.32	0.51	0.45	5

^a^ r: correlation coefficient after nutrient or food group crude intake was transformed to natural logarithm (ln). ^b^ r-adj: correlation coefficient after the nutrient intake or food group intake was adjusted for energy using the residual method. ^c^ r de-att: de-attenuated correlation coefficients after log-transformation and energy adjustment. ^‡^ Percentage of subjects with values out of the limits of agreement.

## Data Availability

Access to data can be requested from the corresponding author.

## Appendix A

**Table A1:** Characteristics of the populations in the reproducibility (*n* = 144) and validity (*n* = 150) studies.

Characteristics of Reproducibility Participants	All (*n* = 144)	Male *n* = 74 (51.4%)	Female *n* = 70 (48.6%)	*p*-Value	Characteristics of Validity Participants	All (*n* = 150)	Male *n* = 79 (52.6%)	Female *n* = 71 (47.4%)	*p*-Value
	Mean ± SD or n (%)	Mean ± SD or n (%)	Mean ± SD or n (%)			Mean ± SD or n (%)	Mean ± SD or n (%)	Mean ± SD or n (%)	
Age (years)	14.42 + 1.69	14.55 + 1.7	14.29 + 1.67	0.34	Age	14.25 + 2.01	14.39 + 2	14.10 + 2.02	0.37
Level of education					Level of education				
Third cycle of primary school	17(11.8%)	9(12.2%)	8(11.4%)	0.23	Third cycle of primary school	22(14.7%)	12(15.2%)	10(14.1%)	0.24
Secondary school	85(59%)	39(52.7%)	46(65.7%)		Secondary school	85(56.7%)	40(50.6%)	45(63.4%)	
High school	42(29.2%)	26(35%)	16(22.9%)		High school	43(28.7%)	27(34.2%)	16(22.5%)	
Weight (kg)	58.2 + 13.2	62.6 + 13.8	53.7 + 10.8	<0.001	Weight (kg)	58.0 + 13.3	61.6 + 14.2	53.9 + 11.2	<0.001
Height (cm)	164.7 + 12.6	169.8 + 12.5	159.2 + 10.1	<0.001	Height (cm)	164.5 + 12.6	164.6 + 13.9	160.5 + 11.2	0.19
z-BMI (kg/m^2^)	21.2 + 3.1	21.5 + 3.3	21.0 + 2.9	0.31	z-BMI (kg/m^2^)	21.1 + 3.2	21.2 + 3.4	21.0 + 3.1	0.85
Optimal	108(74.8%)	55(73%)	54(76.8%)	0.28	Optimal	113(75.3%)	59(74.9%)	54(76.1%)	0.09
Overweight	29(20.3%)	13(17.6%)	16(23.2%)		Overweight	29(19.3%)	13(16.5%)	16(22.5%)	
Obesity	7(4.9%)	7(9.5%)	0		Obesity	8(5.3%)	7(8.9%)	1(1.4%)	
Activity level					Activity level				
Low	46(31.9%)	7(9.5%)	39(55.7%)	<0.001	Low	47(31.3%)	8(10.1%)	39(54.9%)	<0.001
Moderate	65(45.1%)	39(52.7%)	26(37.1%)		Moderate	67(44.7%)	40(50.6%)	27(38.0%)	
High	33(22.9%)	28(37.8%)	5(7.1%)		High	36(24.0%)	30(39.2%)	5(7.0%)	

*p*-values of the mean differences between males and females were calculated using the *t*-test or Mann-Whitney U test for continuous variables and the chi-square test for categorical variables.

**Table A2:** Food group intake of the population in the reproducibility study (*n* = 144).

Food Groups	FFQ1						FFQ2						*p* ^‡^
	M *	Med *	SD *	25	50	75	M	Med	SD	25	50	75	
Sugary beverages (g)	109.9	63.9	146.9	26.8	63.9	152.8	112.8	64.5	124.2	23.9	64.5	167.6	0.05
Fresh fruit (g)	458.1	358.3	451.6	197	358.3	568.1	461.2	407.8	342.4	196	407.8	612.5	0.01
Legumes (g)	25.2	21.5	25.1	11.4	21.5	32.9	26.2	22.7	23	12	22.7	33.1	0.23
Nuts and oil olive (g)	12.8	7.7	14.9	3.2	7.7	18.4	12.2	9.0	11.3	3.6	9.0	19.3	0.56
Vegetables (g)	329.2	241.4	318.2	117.9	241.4	449.2	339.3	278.1	293	144.5	278.1	443.1	0.49
Sauces and processed food (g)	143.5	133.4	89.1	89.1	133.4	174.9	146.8	132.9	70	98.4	132.9	185.8	0.08
Sugar confectionary (g)	83.9	54.9	104.8	25	54.9	92.5	76	56.4	63.5	29.3	56.4	104.3	0.001
Eggs (g)	16.9	7.9	16.2	7.9	7.9	23.7	17.2	13.1	14.4	7.3	13.1	25.8	0.21
Cheese (g)	30	17.2	35.3	5.7	17.2	40	29.2	22.1	28.9	6.5	22.1	41.4	0.1
Pastries (g)	55.7	34.8	74.7	18.5	34.8	57	51.6	39.4	46.8	21.7	39.4	66.4	0.42
Yogurt and milk (g)	233.4	204.2	199.1	86	204.2	308.3	245.4	196.2	219.6	91.1	196.2	339.1	0.10
Potatoes (g)	71.3	47.2	77.4	31.5	47.2	96	70.3	60.7	51.5	33.1	60.7	88.6	0.05
Whole grains (g)	41.7	34.1	40.3	22.6	34.1	52.4	44.2	35.2	37.1	22.8	35.2	57.6	0.01
Refined cereals (g)	112.7	92.4	81.9	53.1	92.4	169.1	114.5	106.6	71.1	62.2	106.6	156.4	0.001
Fish (g)	48.2	31.5	44.3	16.9	31.5	74.6	50.9	37.5	44	20.5	37.5	70.6	0.23
Red meat (g)	81.8	74.6	63.4	33.7	74.6	114.6	85.6	79.9	63.6	41.2	79.9	116	0.48
White meat (g)	53.3	64.5	45.5	21.5	64.5	64.5	57.5	48.7	46.2	26.4	48.7	74.8	0.87
Processed meat (g)	51.3	43.8	37.6	27.8	43.8	67.8	54.4	47.5	36.9	29.2	47.5	70.9	0.03
Animals fats (g)	1.7	0.9	2.7	0	0.9	2	1.9	1	3.2	0	1	2.3	0.50
Vegetable fats (g)	6.3	3.4	8.4	1.1	3.4	6.8	6.1	4.3	7.2	1.7	4.3	7.2	0.09

* Intakes are expressed as the mean (M), standard deviation (SD), median (Med), and interquartile range (P25:P75). ^‡^ *p*-values of the median differences were calculated using the Wilcoxon test.

**Table A3:** Reliability and consistency of food group intake between FFQ1 and FFQ2.

Food Group	ICCs ^1^		Pearson or Spearman Coefficient	
	r ^a^	r-Adjusted ^b^	r ^a^	r-Adjusted ^b^
Sugary Beverages (g)	0.540 (0.413–0.646)	0.459 (0.305–0.591)	0.58	0.59
Fresh fruit (g)	0.310 (0.154–0.450)	0.422 (0.276–0.549)	0.6	0.56
Legumes (g)	0.190 (0.028–0.342)	0.335 (0.168–0.483)	0.42	0.42 *
Nuts and oil olive (g)	0.226 (0.065–0.375)	0.407 (0.254–0.541)	0.48	0.46
Vegetables (g)	0.463 (0.324–0.582)	0.597 (0.477–0.695)	0.68	0.67
Sauces and processed food (g)	0.464 (0.325–0.583)	0.345 (0.191–0.482)	0.34	0.21 *
Sugar confectionary (g)	0.240 (0.08–0.388)	0.419 (0.273–0.545)	0.44	0.42 *
Eggs (g)	0.585 (0.467–0.683)	0.354 (0.189–0.499)	0.3	0.21
Cheese (g)	0.213 (0.052–0.363)	0.112 (−0.07–0.294)	0.6	0.61
Pastries (g)	0.147 (−0.016–0.303)	0.291 (0.133–0.435)	0.33	0.32
Yogurt and milk (g)	0.528 (0.399–0.636)	0.551 (0.422–0.658)	0.62	0.60
Potatoes (g)	0.489 (0.354–0.604)	0.341 (0.186–0.479)	0.5	0.35
Whole grain (g)	0.33 (0.180–0.470)	0.418 (0.266–0.55)	0.49	0.48
Refined cereals (g)	0.376 (0.227–0.508)	0.267 (0.106–0.414)	0.43	0.33
Fish (g)	0.482 (0.347–0.598)	0.449 (0.293–0.582)	0.69	0.69
Red meat (g)	0.511 (0.38–0.622)	0.418 (0.266–0.55)	0.67	0.64
White meat (g)	0.406 (0.261–0.534)	0.238 (0.075–0.389)	0.46	0.36
Processed meat (g)	0.664 (0.562–0.747)	0.508 (0.375–0.621)	0.74	0.67 *
Animals fats (g)	0.270 (0.112–0.415)	0.407 (0.181–0.592)	0.43	0.47
Vegetable fats (g)	0.201 (0.039–0.352)	0.308 (0.135–0.463)	0.3	0.28

FFQ1 vs. FFQ2 after 10 months. ^1^ ICC: intra-class correlation coefficient. ^a^ r: ICC after nutrient or food group crude intake was transformed to the natural logarithm (ln). ^b^ r-adjusted: after nutrient intake or food group intake was adjusted for energy using the residual method. * Pearson correlation.

**Table A4:** Agreement percentages for nutrients and food groups between FFQ1 and FFQ2 and misclassification (*n* = 144).

Nutrients	Misclassification ^1^		Agreement Percentages ^2^	
	N	%	N	%
Energy (kcal)	6	4.2	118	81.9
Total protein (g)	8	5.6	114	79.2
Animal protein (g)	6	4.2	114	79.2
Vegetable protein (g)	9	6.3	108	75
Total fats (g)	10	6.9	107	74.3
MUFAs (g)	9	6.3	105	72.9
PUFAs (g)	7	4.9	106	73.9
SFAs (g)	11	7.6	110	76.4
Cholesterol (mg)	8	5.6	110	76.4
Carbohydrates (g)	8	5.6	115	79.9
Total fiber (g)	7	4.9	114	79.2
Iodine (µg)	9	6.3	110	76.4
Sodium (mg)	10	6.9	111	77.1
Potassium (mg)	7	4.9	122	84.7
Calcium (mg)	13	9.0	111	77.1
Magnesium (mg)	11	7.6	111	77.1
Phosphorus (mg)	7	4.9	113	78.5
Iron (mg)	15	10.4	103	71.5
Zinc (mg)	7	4.9	110	76.4
Selenium (µg)	8	5.6	112	77.8
Vitamin B1 (mg)	7	4.9	123	85.4
Vitamin B2 (mg)	6	4.2	116	80.6
Vitamin B6 (mg)	8	5.6	121	81
Vitamin B12 (µg)	6	4.2	114	79.2
Vitamin B9 or Folate (µg)	5	3.5	117	81.3
Vitamin B3 or niacin (mg)	5	3.5	114	79.2
Vitamin C (mg)	4	2.8	117	81.3
Vitamin A (µg)	6	4.2	114	79.2
Vitamin D (µg)	12	8.3	103	71.5
Vitamin E (mg)	8	5.5	110	76.4
Nutrients Average	8.1	5.6	112.6	78.2
Sugary beverages	3	2.1	119	82.6
Fresh fruit	4	2.8	127	88.2
Legumes	6	4.2	115	79.9
Nuts and oil olive	4	2.8	119	82.64
Vegetables	1	0.7	127	88.2
Sauces and processed food	7	4.9	109	75.7
Sugar confectionary	6	4.2	106	73.6
Eggs	3	2.1	124	86.1
Cheese	3	2.1	119	82.6
Pastries	1	0.7	118	81.9
Yogurt and milk	1	0.7	121	84.03
Potatoes	4	2.8	114	79.2
Whole grain	1	0.7	108	75.0
Refined cereals	4	2.8	114	79.2
Fish	0	0.0	127	88.2
Red meat	1	0.7	114	79.2
White meat	0	0.0	129	89.6
Processed meat	4	2.8	126	87.5
Animals fats	4	2.8	110	76.4
Vegetables fats	6	4.2	114	79.2
Food group Average	3	2.2	118	81.9

^1^ Misclassification: percentage of participants belonging to the opposite quartile. ^2^ Percentage of participant belonging to the same or an adjacent quartile.

**Table A5:** Agreement percentages between FFQ2 and 24H-DR and misclassification (*n* = 150).

Nutrients	Misclassification ^1^		Agreement Percentages ^2^	
	N	%	N	%
Energy (kcal)	14	9.3	110	72.9
SFAs (g)	14	9.3	99	65.6
Sodium (mg)	13	8.6	106	70.2
Cholesterol (mg)	8	5.3	99	65.6
Iodine (µg)	14	9.3	107	70.9
Potassium (mg)	11	7.3	106	70.2
Calcium (mg)	15	9.9	111	73.5
Magnesium (mg)	9	6.0	114	75.5
Phosphorus (mg)	15	9.9	113	74.8
Iron (mg)	8	5.3	113	74.8
Selenium (µg)	11	7.3	117	77.5
Zinc (mg)	14	9.3	103	68.2
Vitamin B1 (mg)	9	6.0	112	74.2
Vitamin B2 (mg)	10	6.6	116	76.8
Vitamin B6 (mg)	6	3.9	115	76.2
Vitamin B12 (µg)	9	6.0	105	69.5
Vitamin B9 or Folate (µg)	6	4.0	113	74.8
Vitamin B3 or Niacin (mg)	7	4.6	107	70.9
Vitamin C (mg)	6	4.0	126	83.4
Vitamin A (µg)	7	4.6	111	73.5
Vitamin D (µg)	13	8.6	106	70.2
Vitamin E (mg)	13	8.6	100	66.2
Carbohydrates (g)	11	7.3	108	71.5
Total protein (g)	11	7.3	109	72.2
Total fats (g)	16	10.6	108	71.5
PUFAs (g)	13	8.6	104	68.9
MUFAs (g)	14	9.3	112	74.2
Total fiber (g)	5	3.3	114	75.5
Average	10.8	7.1	109.4	72.5

^1^ Misclassification: percentage of participants belonging to the opposite quartile. ^2^ Percentage of participants belonging to the same or an adjacent quartile.
